# A prospective, multicenter, real-world effectiveness and safety study of high molecular weight sodium hyaluronate for interstitial cystitis/bladder pain syndrome

**DOI:** 10.1007/s11255-026-05035-1

**Published:** 2026-02-05

**Authors:** Robert Stoica, Jose Medina-Polo, Rustom P. Manecksha, Christine Kolb, Melanie Emmeluth, Hans Christian Kuhl, Tarek Hassan, Claus R. Riedl

**Affiliations:** 1 Fundeni Clinical Institute, University of Medicine and Pharmacy Carol Davila, Bucharest, Romania; 2 Department of Urology, Hospital Universitario 12 de Octubre and UCM, Madrid, Spain; 3 Department of Surgery, Tallaght University Hospital, Dublin, Ireland; 4 School of Medicine, Trinity College Dublin, Dublin, Ireland; 5 MEDA Pharma GmbH and Co. KG (A Viatris Company), Bad Homburg, Germany; 6 Viatris Inc, Canonsburg, PA USA; 7 Department of Urology, LKH Baden, Waltersdorfer Straße 75, 2500 Baden, Austria

**Keywords:** Interstitial cystitis, Intravesical sodium hyaluronate, Performance, Real-world, Safety, Bladder pain

## Abstract

**Purpose:**

The aim of the present study was to evaluate the performance and safety of high molecular weight (HMW) sodium hyaluronate (40 mg/50 mL) for interstitial cystitis/bladder pain syndrome (IC/BPS) in real-world clinical practice.

**Methods:**

This prospective, multicenter European study was conducted in patients with the clinical diagnosis of IC/BPS. Participants received weekly intravesical instillations for 12 weeks. The primary endpoint was responder rate at end of treatment/week 12, defined as any improvement in IC/BPS symptoms on the 7-point Patient Global Assessment (PGA) scale. Secondary endpoints included changes in bladder symptoms and quality of life assessed by visual analog scales (VAS), questionnaires, and voiding diaries at week 12 and 24.

**Results:**

Seventy-one (N = 74) patients enrolled were part of the full analysis set (mean [SD] age: 51.8 [16.9] years; 98.6% female) and 73/74 patients were in the safety set (mean [SD] age: 51.9 [17.2] years; 98.6% female). Total responder rate was 90.1% (90% CI: 82.3, 95.3) at week 12 and 78.9% (90% CI: 69.4, 86.5) at week 24. Significant improvements from baseline were observed in VAS scores for urinary urgency and bladder pain at week 12 (–42.0; –39.3) and week 24 (–49.2; –49.2). Quality of life scores also improved significantly (+ 17.1; + 27.2, all *P* < .0001). Twenty patients (27.4%) reported 37 adverse events (AEs), including seven treatment-related AEs (incidents). No treatment-related serious AEs occurred and all incidents were resolved.

**Conclusions:**

Intravesical HMW sodium hyaluronate improved IC/BPS symptoms and quality of life through week 24 in most patients with a favorable safety profile.

**Supplementary Information:**

The online version contains supplementary material available at 10.1007/s11255-026-05035-1.

## Introduction

Interstitial cystitis (IC)/Bladder Pain Syndrome (BPS) is a chronic condition of the urinary bladder characterized by pain, increased urinary frequency, urgency, and reduced voiding volume [1, 2]. The etiology of IC/BPS is heterogeneous, and its pathophysiology remains incompletely understood. Historically, disruption of the urothelial glycosaminoglycan (GAG) layer was proposed as a central mechanism contributing to urothelial barrier dysfunction and urinary symptoms in a subset of patients [3–5]. Agents such as hyaluronic acid, chondroitin sulphate, and heparin can augment or stabilize the uroepithelial barrier and have demonstrated efficacy in relieving symptoms in patients with IC/BPS [6].

More recent findings, however, suggest that this concept oversimplifies a complex process. Evidence indicates that impaired urothelial maturation, instability of the urothelial barrier, and disturbed GAG metabolism lead to excessive shedding of glycosaminoglycans in patients with IC/BPS compared to controls [7, 8]. Increased urinary hyaluronic acid excretion and overexpression of hyaluronan synthase 3 (HAS3) further support the view that what has historically been described as GAG layer disruption likely reflects altered synthesis, shedding, and differentiation rather than a simple depletion of a protective layer [9].

The urothelial barrier is critical in protecting underlying tissues from urinary solutes and pathogens. Intravesical administration of high molecular weight (HMW; > 1000 kDa) sodium hyaluronate, a sodium salt of hyaluronic acid, has been investigated as a therapeutic strategy in IC/BPS [10]. Rather than replenishing or rebuilding a damaged GAG layer, intravesical HMW sodium hyaluronate is thought to provide a hydrophilic surface coating that reinforces urothelial barrier function, while potentially modulating inflammatory signaling and facilitating epithelial repair [11, 12]. These mechanisms are biologically plausible. However, it must be underscored that current clinical data do not establish or confirm a specific mode of action. The efficacy and safety of intravesical HMW sodium hyaluronate has been demonstrated in both uncontrolled and placebo-controlled studies, with symptom remission reported in 65 to 85% of patients with IC/BPS [13–18].

The present study was conducted as a prospective, multicenter, real-world evaluation of the effectiveness and safety of intravesical HMW sodium hyaluronate. By complementing existing randomized trial evidence, this study aims to provide clinically relevant insights into the utility of this intervention in routine practice across diverse patient populations and treatment settings.

## Methods

### Study design

This prospective, multicenter, non-interventional European study was conducted between October 2022 and July 2024 across 13 sites in Austria, Ireland, Romania, and Spain. Eligible patients had a clinical diagnosis of IC/BPS confirmed using standardized measures consistent with ESSIC criteria.These criteria include chronic bladder pain (≥ 6 months), bladder-related pressure/discomfort, ≥ 1 accompanying intermittent or persistent lower urinary tract symptom (e.g., urgency, frequency, or nocturia in the past 6 months), a Bladder Pain/ Interstitial Cystitis Symptom Score (BPIC-SS) > 18 prior to first treatment, and the exclusion of confounding conditions. Cystoscopy is not mandatory for the diagnosis of IC/BPS, but it plays a critical role in excluding confounding conditions and ruling out malignancy [2]. Importantly, cystoscopy is indispensable for identifying Hunner lesion disease, which represents a distinct IC/BPS phenotype with a well-defined therapeutic approach. For this reason, all patients in the present study underwent cystoscopy prior to initiation of therapy, if in accordance with routine clinical practice at the site.

Following enrollment, participants received weekly intravesical instillations of HMW sodium hyaluronate under standard of care for 12 weeks. Clinical assessments were performed at end of treatment (EoT) or week 12, whichever occurred later, and at a follow-up visit scheduled around 24 weeks (six months) after treatment initiation, regardless of treatment duration. Outcomes included responder rate based on the Patient Global Assessment (PGA) scale, changes in bladder symptoms and quality of life measured by validated questionnaires, visual analog scales (VAS), and voiding diaries, as well as safety evaluations through adverse event monitoring.The study was approved by local research ethics committees at all participating sites and countries and adhered to the Declaration of Helsinki and the DIN ISO 14155 standard for clinical investigations of medical devices. All patients were required to provide written informed consent before study entry, and study participation was voluntary.

### Study participants

Seventy-four adult patients were planned for enrollment, accounting for an anticipated dropout rate of approximately 10% prior to EoT/week 12. This estimation was based on previous studies reporting a minimum 45% responder rate for the primary endpoint [15]. It was further estimated that only half of the patients reporting slight improvement in IC/BPS would meet the responder definition when additional criteria such as willingness for retreatment and a self-reported positive impact of treatment on quality of life (QoL) were applied. Under these assumptions, a minimum of 67 evaluable patients and an additional 7 patients to compensate for potential dropouts were required to estimate the responder rate with a two-sided 90% confidence interval and a 10% margin of error.

### Study criteria

-  ≥ 18 years of age
- Naïve to prior prescription of Cystistat® (Mylan Institutional, Inverin, Co. Galway, Ireland), a clear colorless sterile solution presented in a 50 mL glass vial containing 40 mg sodium hyaluronate
- Clinically diagnosis of IC/BPS (preferably using European Society for the Study of Interstitial Cystitis [European Society for the Study of Interstitial Cystitis]), including cystoscopy to confirm diagnosis of IC/BPS (if in accordance with routine clinical practice at the site)
-  ≥ 6 months of bladder pain/discomfort (e.g., constant bladder pain/discomfort or bladder pain/discomfort when voiding or as a burning sensation between voids as the bladder fills with urine)
-  ≥ 1 accompanying intermittent or persistent lower urinary tract symptoms, such as urinary frequency, urgency, or nocturia during the previous 6 months, and a a
- Bladder Pain/Interstitial Cystitis Symptom Score (BPIC-SS) ≥ 18 prior to first treatment.

Patients were excluded from the study if they had a known hypersensitivity to HMW sodium hyaluronate, prior GAG substitution therapy within two years, history of fulguration or resection of Hunner’s lesions, pregnancy or intent to become pregnant, or diagnosis of recurrent urinary tract infection (UTI) or overactive bladder. Therapeutic bladder hydrodistension (under regional/general anesthesia) should not have been performed within 1 year preceding enrolment and throughout the duration of the study, if possible. Patients could withdraw at any point without prejudice. Reasons for study withdrawal were recorded.

### Study endpoints and measures

Study endpoints were assessed in the full analysis set (FAS), comprising patients who received ≥ 1 HMW sodium hyaluronate instillation and had ≥ 1 post-baseline assessment. Safety was assessed in the safety set (SAF), i.e., patients who received ≥ 1 HMW sodium hyaluronate instillation.

The primary study endpoint was the responder rate at EoT or week 12 (whichever occurred later), defined by improvement in IC/BPS symptoms on the 7-point Patient Global Assessment (PGA) scale, i.e., evaluated as markedly, moderately, or slightly improved. In the case of slightly improved condition, only patients who voted favorably for retreatment and reported a positive effect of treatment on QoL were considered as responders. The PGA is a patient-reported outcome measure rating IC/BPS symptom change from markedly worse to markedly improved on a 7-point scale [19] (Supplemental Fig. 1).

Secondary endpoints included: changes from baseline in (1) bladder pain, (2) urinary urgency, and (3) QoL on the visual analog scale (VAS). Patients rated bladder symptoms (pain and urinary urgency) and QoL on a 0–100 mm visual analog scale at baseline, EoT/week 12, and week 24. The VAS is a widely used measurement instrument in clinical studies for IC/BPS, with high reproducibility [19, 20]. A score of zero indicated no bladder symptoms/ or worst imaginable QoL, whereas 100 indicated worst possible bladder symptoms or best imaginable QoL, based on the domain it measured. Other secondary endpoints included changes in BPIC-SS scores, first voided morning volume, daytime and nighttime urinary frequency and voiding volume, occurrence, and number of flares until week 24, changes in the BPIC-SS questionnaire in case of a flare, and use of concomitant pain medication. Responder rates, as defined for the primary endpoint, were also assessed at week 24. Responder rates using a stricter responder definition, i.e., patients who reported moderate or marked improvement on the PGA scale, were also assessed at identical time points—EoT/week 12 and week 24.

Safety assessments included all adverse events (AEs), product malfunctions, and use errors reported through week 24. AEs were categorized by severity and relatedness. Events deemed related to treatment were classified as incidents. Any AEs reported prior to instillations were recorded as unrelated to treatment. Patients were also monitored for side effects mentioned in the instructions for use, such as local rash/itching, bladder/urethral pain and discomfort, or UTI.

### Statistical analysis

All analyses were exploratory and descriptive. Categorical variables were summarized using frequencies and percentages, while continuous variables were reported as means with standard deviations (SD), medians, range, and quartiles. To assess the effect of covariates such as age, treatment duration, and number of instillations on being a responder at EoT/week 12, a logistic regression analysis was performed. Results were reported as odds ratios (OR) and 95% confidence intervals (CI). Lastly, a subgroup analysis was also performed on a group of patients who had ≥ 4 HMW sodium hyaluronate instillations within the first 25 days of treatment, consistent with the device’s instructions for use (IFU). All statistical analyses were performed using SAS version 9.4 (SAS Institute Inc., Cary, NC, USA).

## Results

### Patient characteristics

Of the 74 patients with IC/BPS enrolled in the study, 71 patients were included in the FAS (mean [SD] age: 51.8 [16.9] years, 98.6% female, 91.5% Caucasian) and 73 in the SAF (mean [SD] age: 51.9 [17.2] years, 98.6% female, 91.8% Caucasian; Table 1). Fifty-seven patients (78.1%) were diagnosed with IC/BPS using ESSIC criteria. Overall, 68 patients (93.2%) completed the study. Four patients withdrew consent, and two were lost to follow-up (Supplemental Fig. 2). Four patients (5.5%) reported having relevant renal/urinary disorders prior to HMW sodium hyaluronate instillation including urinary urgency (2.7%) and lower UTI symptoms (1.4%; Supplemental Table 1). One patient (1.4%) presented with Hunner’s lesions without prior fulguration or resection. Nine patients (12.3%) reported prior use of IC/BPS medications before enrollment, while 21 patients (28.8%) reported using IC/BPS medications before and after enrollment. Urological therapies were the most frequently used IC/BPS medications before enrollment (8 patients, 11.0%), and both prior to and following enrollment (18 patients, 24.7%). Twenty-one patients (28.8%) reported use of at least one pain medication before and after enrollment, most commonly analgesics and neurologicals, each used by seven patients (9.6%).

**Table 1:** Patient demographics and treatment compliance

Variables	FAS (N = 71)	SAF (N = 73)
Age, (year)		
Mean (SD)	51.8 (16.9)	51.9 (17.2)
Median (IQR)	49.0 (39.0, 66.0)	49.0 (39.0, 66.0)
Gender, (n [%])		
Female	70 (98.6)	72 (98.6)
Male	1 (1.4)	1 (1.4)
Race/Ethnicity, (n [%])		
Caucasian	65 (91.5)	67 (91.8)
African	1 (1.4)	1 (1.4)
Hispanic	5 (7.0)	5 (6.8)
Other^1^	0 (0.0)	0 (0.0)
Treatment Duration, (week)		
Mean (SD)	10.8 (2.2)	10.5 (2.8)
Median (IQR)	11.0 (10.0, 11.9)	11.0 (10.0, 11.6)
Number of sodium hyaluronate instillations per patient until week 12, (n [%])		
1–5	3 (4.2)	5 (6.8)
6	19 (26.8)	19 (26.0)
7	3 (4.2)	3 (4.1)
8	1 (1.4)	1 (1.4)
9	3 (4.2)	3 (4.1)
10	7 (9.9)	7 (9.6)
11	2 (2.8)	2 (2.7)
12	33 (46.5)	33 (45.2)
Patients with ≥ 4 sodium hyaluronate instillations in first 25 days of treatment, (n [%])		
Yes	47 (66.2)	47 (64.4)
No	24 (33.8)	26 (35.6)

Abbreviations: IQR, Interquartile range; FAS, Full analysis set; n, Number of patients in group; N, Total number of patients in the population; SAF, Safety set; SD, Standard deviation

^1^Other race/ethnicities included American, Asian, Unknown, and Other

### Treatment compliance

During the study, most of the patients (n = 68; 95.8%) had received between six and twelve HMW sodium hyaluronate instillations until EoT/week 12, with 33 patients (46.5%) receiving 12 HMW sodium hyaluronate instillations (Table 1). A substantial proportion of patients received ≥ 4 instillations during the first 25 days of treatment (66.2% in FAS; 64.4% in SAF). On average, patients continued treatment for 10.8 weeks (FAS) and 10.5 weeks (SAF), with a median treatment duration of 11 weeks (Table 1). HMW sodium hyaluronate showed good compliance and acceptability, with no interruptions or addition of other medications, including pain medications that were permitted in case of a flare-up.

## Performance outcomes

### Primary analysis

At EoT/week 12, the PGA responder rate was 90.1% (n = 64; 90% CI: 82.3, 95.3). Specifically, 32 patients (45.1%) reported that IC/BPS had markedly improved, 20 patients (28.2%) reported moderate improvement, and 13 patients (18.3%) reported slight improvement. Of those with slight improvement (n = 13), 12 patients (92.3%) endorsed a positive effect on life from HMW sodium hyaluronate use and considered repeating the treatment. None of the patients reported a worsening of their IC/BPS condition and only three patients (4.2%) reported no change in IC/BPS from treatment (Table 2).

**Table 2:** PGA and responder rate at EOT/Week 12 and Week 24 in FAS (N = 71)

Parameter	EoT/Week 12	Week 24
Responder rate^1^		
n (%)	64 (90.1)	56 (78.9)
90% CI	82.3, 95.3	69.4, 86.5
Overall change in IC/BPS condition on PGA scale (rating), (n [%])		
Markedly improved	32 (45.1)	34 (47.9)
Moderately improved	20 (28.2)	15 (21.1)
Slightly improved	13 (18.3)	9 (12.7)
No change	3 (4.2)	3 (4.2)
Slightly worse	0 (0.0)	2 (2.8)
Moderately worse	0 (0.0)	0 (0.0)
Markedly worse	0 (0.0)	3 (4.2)
Missing	3 (4.2)	5 (7.0)
In case of slightly improved, did therapy have a positive effect and would you n = 13 n = 9 undergo this treatment again?		
Yes	12 (92.3)	7 (77.8)
No	1 (7.7)	2 (22.2)

Abbreviations: CI, Confidence interval; EoT, End of treatment; FAS, Full analysis set; IC/BPS, Interstitial cystitis/bladder pain syndrome; n, Number of patients in group; N, Total number of patients in the population; PGA, Patient global assessment

^1^ Responder definition: a responder was defined as a patient who has experienced a marked, moderate, or slight improvement on the 7-point Patient Global Assessment. In case of slight improvement, patients were only considered as responder if the question “Did therapy have a positive effect on your life, and would you undergo this treatment again?” was answered with “Yes”

Among the subgroup of patients with ≥ 4 HMW sodium hyaluronate instillations in the first 25 days of treatment (N = 47), the responder rate was 91.5% (n = 43; 90% CI: 81.6, 97.0) at EOT/week 12. This was slightly higher than that of the primary analysis in the FAS. Twenty-five patients (53.2%) reported that IC/BPS had markedly improved, 15 patients (31.9%) reported moderate improvement, and four patients (8.5%) reported slight improvement. No patients reported worsening of their IC/BPS symptoms.

### Secondary analyses

**Fig. 1:**
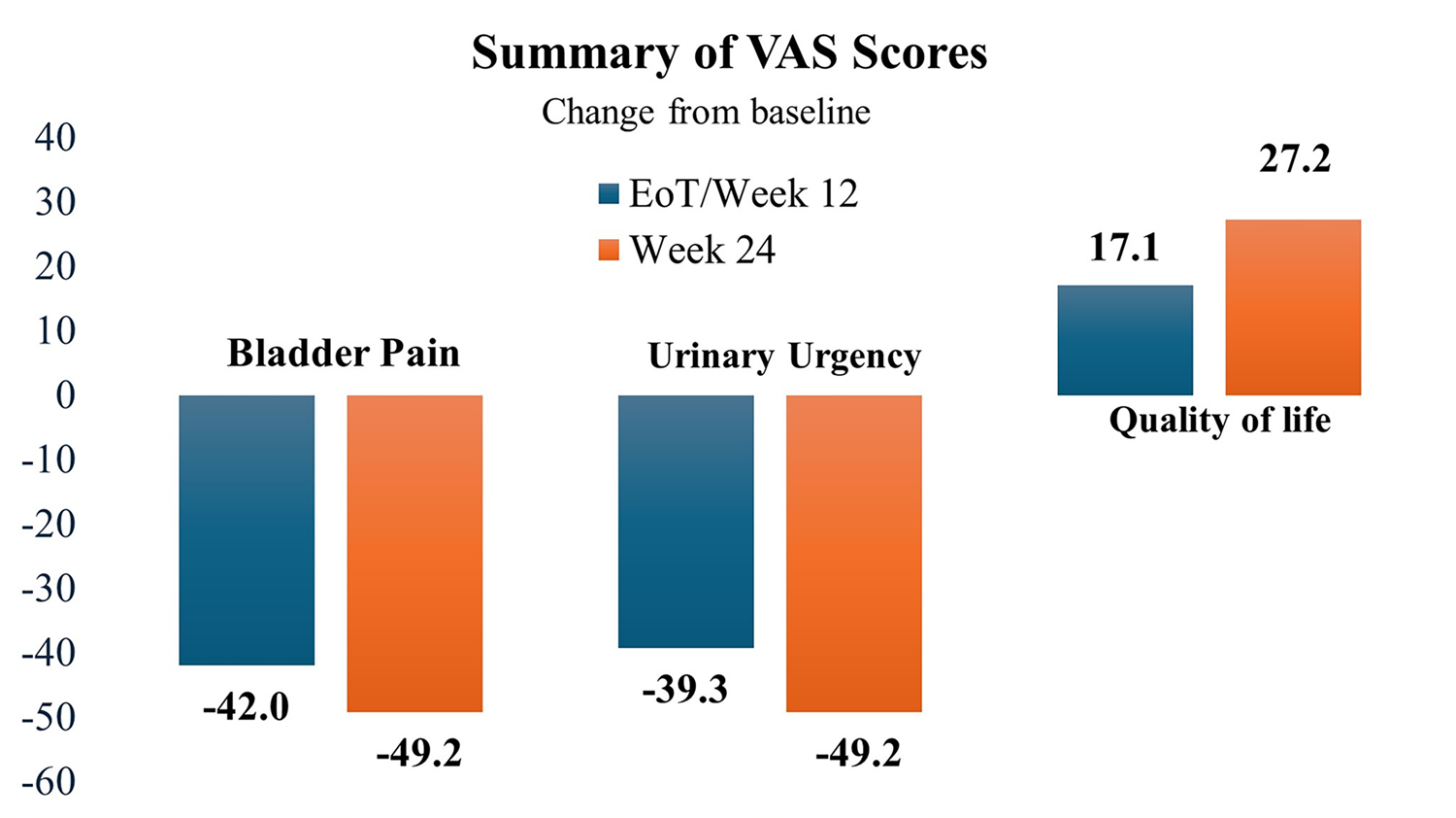
Change from baseline to EoT/week 12 and week 24 in VAS scores in FAS (N = 71) Note: Change values at EoT/week 12 for bladder symptoms and quality of life were significant at *P* < .0001. Abbreviations: EoT, End of treatment; FAS, Full analysis set; N, Total number of patients in the population; VAS, Visual analog scale

A comparison of self-reported VAS scores for bladder pain, urinary urgency and QoL at EoT/week 12 and week 24 versus baseline showed significant improvements with HMW sodium hyaluronate treatment (Fig. 1). The mean (SD) VAS score for bladder pain was significantly lower at EoT/week 12 (33.1 [27.1]) compared to baseline (74.7 [18.7]; *P* < 0.0001) and decreased further to 25.8 (25.3) at week 24 (*P* < 0.0001). Urinary urgency VAS scores also decreased from 75.2 (21.7) at baseline to 36.7 (29.3) at EoT/week 12 and further reduced to 26.7 (29.8) at week 24 (all at *P* < 0.0001). Lastly, the mean (SD) scores for QoL improved from baseline (53.1 [28.9]) to 69.9 (24.7) at EoT/week 12 (*P* < 0.0001) and further increasing at week 24 to 79.8 (20.3; *P* < 0.0001; Fig. 1).

At week 24, the responder rate was 78.9% (n = 56; 90% CI: 69.4, 86.5); 34 (47.9%) patients reported a marked improvement in their IC/BPS condition, 15 patients (21.1%) reported moderate improvement, and nine patients (12.7%) reported slight improvement of whom seven patients (77.8%) perceived a positive effect on life from HMW sodium hyaluronate treatment and would consider repeat treatment. Importantly, at week 24 of HMW sodium hyaluronate instillation, only five (7.0%) patients reported worsening of their IC/BPS symptoms (Table 2).

The mean (SD) BPIC-SS score decreased to 15.6 (8.5) at EoT/week 12 from 29.3 (4.6) at baseline. At week 24, a further decline to 14.3 (9.2) was observed. Mean (SD) BPIC-SS score reductions from baseline were 13.8 (8.6) at EoT/week 12 and 15.3 (9.2) at week 24 (both *P* < 0.0001; Table 3). The mean first morning voiding volume increased from baseline to EoT/week 12 and week 24 (184.2 mL to 197.5 mL to 222.4 mL). Total number of daytime and night-time voids also decreased from baseline to EoT/week 12 (10.4 to 8.5, *P* < 0.0001; and 2.9 to 2.0, *P* = 0.0007) and week 24 (10.4 to 7.0, *P* < 0.0001; and 2.9 to 1.5, *P* = 0.0087), respectively (Table 3). Because of the large number of missing values in these data (> 40%), these results are to be interpreted with caution.

**Table 3:** Summary of other secondary endpoints in FAS (N = 71)

Parameter	EoT/Week 12	Week 24
First morning void volume (mL), Mean (SD) ^1^	197.5 (113.7)	222.4 (88.5)
Change from baseline in morning voiding volume (mL), Mean (SD) ^±^	+ 24.0 (122.8)	+ 20.9 (90.6)
Total daytime voiding volume (mL), Mean (SD) ^1^	1247.8 (551.2)	1272.3 (473.7)
Change from baseline in total daytime voiding volume (mL), Mean (SD) ^1^	+ 59.0 (376.7)	+ 89.4 (364.3)
Total nighttime voiding volume (mL), Mean (SD) ±	272.7 (204.6)	236.4 (226.7)
Change from baseline in total nighttime voiding volume (mL), Mean (SD) ^1^	−68.7 (204.0) *	−56.4 (178.0)
Total number of voids during daytime, Mean (SD) ^±^	8.5 (5.1)	7.0 (2.9)
Change from baseline in total number of voids during daytime, Mean (SD) ^1^	−2.9 (3.4) **	−3.0 (3.1) **
Total number of voids during nighttime, Mean (SD) ±	2.0 (1.9)	1.5 (2.1)
Change from baseline in total number of voids during nighttime, Mean (SD)^1^	−1.1 (1.8) **	−1.1 (2.2) *
BPIC-SS score, Mean (SD)	15.6 (8.5)	14.3 (9.2)
Change from baseline in BPIC-SS score, Mean (SD)	−13.8 (8.6) **	−15.3 (9.2) **
Responder rate using alternative definition ^2^		
n (%)	52 (73.2)	49 (69.0)
90% CI	63.3, 81.7	58.8, 78.0

**Abbreviations:** BPIC-SS, Bladder Pain Interstitial Cystitis – Symptom Score; EoT, End of treatment; FAS, Full analysis set; mL, Milliliter; n, Number of patients in group; N, Total number of patients in the population; SD, Standard

^*^Significant at *P* < .05

^**^ Significant at *P* < .001

^1^Data on > 40% of patients were missing. Missing data was not imputed. Results must be interpreted with caution

^2^Alternative responder definition: a responder was defined as a patient who has experienced a moderate or marked improvement on the 7-point Patient Global Assessment

Using the stricter, alternative responder definition (moderate or marked improvement on PGA scale), rates at EoT/week 12 were 73.2% (n = 52; 90% CI: 63.3, 81.7) and remained stable at week 24 at 69.0% (n = 49; 90% CI: 58.8, 78.0; Table 3). Thirteen patients (17.8%) reported concomitant pain medication use, primarily neurological medications (6 patients; 8.2%) followed by analgesics (5 patients; 6.8%); paracetamol (2 patients; 2.7%) was the most frequently used concomitant pain medication (Supplemental Table 2). No symptom flares were reported by patients. Six (8.2%) patients withdrew from the study prematurely for reasons unrelated to treatment.

The logistic regression analysis did not demonstrate any statistically significant associations between responder status and age (OR = 0.9 [95% CI: 0.9, 1.0, *P* = 0.0834]), treatment duration (OR = 1.1 [95% CI:1.0, 1.2, *P* = 0.0932]), or number of instillations (OR = 1.3 [95% CI: 0.8, 2.2, *P* = 0.2539]).

## Safety outcomes

In this study, HMW sodium hyaluronate use was well tolerated. Five patients (6.8%) experienced seven treatment-related AEs (incidents). These included five incidents of special interest reported by five patients: four patients (5.5%) reported four mild incidents (Grade 1 severity) of bladder pain/urethral pain or discomfort, and one patient (1.4%) reported a UTI (Grade 2 severity). No Grade 3 severity incidents were reported. All seven incidents were resolved. Overall, 20 patients (27.4%) reported 37 AEs, of which 30 AEs in 17 patients (23.3%) were classified as non-treatment-related AEs. One patient (1.4%) experienced a serious non-treatment-related AE (fever). In total, no serious AEs leading to treatment discontinuation occurred with HMW sodium hyaluronate treatment (Table 4).

**Table 4:** Overall summary of adverse events (AEs) in SAF

Adverse Events (AEs), n (%)	SAF (N = 73)	Events (n)
AEs	20 (27.4)	37
Non-related AEs	17 (23.3)	30
Incidents^1^	5 (6.8)	7
Related AEs of special interest	5 (6.8)	5
Allergic reaction (local rash and/or itching)	0 (0.0)	0
Bladder/urethral pain or discomfort	4 (5.5)	4
UTI	1 (1.4)	1
Serious AEs/incidents	1 (1.4)	1
Non-related AEs	1 (1.4)	1
Incidents	0 (0.0)	0
Discontinuations due to serious AEs/incidents	0 (0.0)	0
Product Quality Complaints only or with AE	0 (0.0)	0

Abbreviations: n, Number of patients in group; N, Total number of patients in the population; SAF, Safety set; UTI, Urinary tract infection

^1^All AEs judged by either the reporting investigator or the sponsor as having a reasonable causal relationship to intravesical sodium hyaluronate treatment were designated as incidents

## Discussion

The first evidence of intravesical hyaluronic acid efficacy in IC/BPS was by Morales et al. in 1996, who reported a 71% symptom response in 25 patients after six instillations [15]. Subsequent controlled and uncontrolled studies have demonstrated efficacy rates ranging from 30 to 89% [14–18, 21–29] (Supplemental Table 3).

This prospective, multicenter real-world study assessed the safety and effectiveness of HMW sodium hyaluronate instillation delivered in routine clinical practice across multiple centers, without the restrictions of a randomized controlled trial. Intravesical HMW sodium hyaluronate performed consistently with and exceeded previous study ranges. Among unselected IC/BPS patients, including those with comorbidities and concomitant therapies, 90% were responders at week 12, with 73% reporting moderate-to-marked improvement. Benefits were durable, with 79% maintained improvement at week 24. Treatment was well tolerated, with minimal side effects and good compliance. These findings confirm the effectiveness of HMW sodium hyaluronate in routine practice and support the hypothesis that urothelial barrier dysfunction linked to altered GAG turnover, the cause of IC/BPS in a subset of patients, can be addressed through intravesical substitution. Rather than replenishing a depleted layer, HMW sodium hyaluronate provides a hydrophilic coating, reinforces the barrier, and may modulate inflammatory signaling and repair. Secondary endpoints showed significant improvements in bladder pain, urgency, and QoL VAS scores at weeks 12 and 24, exceeding the Minimally Important Clinical Difference threshold [30].

Despite accumulating evidence for HMW sodium hyaluronate’s efficacy in IC/BPS symptom relief, several uncertainties remain. Current recommendations for instillation frequency, dosing, and dwell time are largely empirical, with limited evaluation of alternative regimens. Some studies report improved outcomes with extended treatment, while others find no differences between six to nine instillations or > 9 [24]. Comparisons are hindered by the absence of a standardized definition of treatment response [23].

Clinical investigations have primarily utilized HMW hyaluronic acid, but it remains unclear whether medium (500–1000 kDa) or low molecular weight (< 500 kDa) forms exhibit comparable efficacy. Although HMW hyaluronic acid offers superior water-binding capacity and barrier function, lower molecular weight forms may achieve more effective receptor-mediated anti-inflammatory activity (via CD44, I-CAM, RHAMM/CD168, TLR2/4, β-defensin 2). Notably, HMW hyaluronic acid undergoes partial degradation to smaller forms, potentially enabling similar receptor interactions [11, 12].

Another challenge is identifying IC/BPS subgroups most likely to benefit. Higher pre-treatment bladder capacity and elevated baseline pain VAS scores were associated with greater therapeutic response [22]. Recent consensus has emphasized the importance of phenotyping IC/BPS into bladder-centric and non-bladder-centric subtypes. Bladder-centric patients, characterized by reduced functional bladder capacity and persistent urinary symptoms [31], appear to derive the greatest benefit from intravesical glycosaminoglycan (GAG) substitution therapies. In contrast, non-bladder-centric patients, who often present with widespread pain syndromes and/or pelvic floor dysfunction [31], may be less responsive to bladder-directed interventions. The inclusion of such heterogeneous patient groups in earlier studies may have contributed to variability and confounding in reported efficacy outcomes.

In our study, the IC/BPS cohort appears predominantly bladder-centric based on the observed symptom profile. This positioning is clinically relevant, as it identifies the subgroup most likely to benefit from intravesical therapies. By focusing on this phenotype, our findings provide clearer external validation of therapeutic effectiveness in routine practice and reinforce the rationale for targeted bladder-directed management strategies in IC/BPS. In addition, treatment-related adverse events were infrequent, mild, and transient, with no product-related quality issues.

Limitations include absence of comparator arms, missing incompleteness of secondary endpoint data, and reliance on self-reported measures. Prior therapeutic bladder hydrodistension was not controlled for and may have confounded outcomes. Only one patient (1.4%) presented with Hunner’s lesions without prior fulguration or resection. This minimal representation precluded meaningful subgroup analysis by lesion status, as results would lack statistical power and generalizability. In addition, based on our clinical experience, most patients with Hunner’s lesion disease do not respond well to GAG substitution therapy. Therefore, we did not pursue such analyses and acknowledge this as a limitation, warranting evaluation in larger, phenotype-balanced cohorts.

## Conclusions

This real-world, non-interventional study of 74 patients demonstrated significant IC/BPS symptom improvements by week 12, with sustained benefits through week 24. Findings corroborate prior efficacy and safety data and validate HMW sodium hyaluronate in routine practice. Importantly, this multicenter study encompassed diverse practice settings, from large public university hospitals serving heterogeneous populations to smaller private clinics with specialized patient bases, thereby reflecting real-world variability. Designed to complement prior uncontrolled or placebo-controlled trials, it confirms the benefit–risk profile of HMW sodium hyaluronate under routine European clinical conditions. Phenotyping may further optimize outcomes by identifying those subsets of IC/BPS patients most likely to respond.

## Supplementary Information

Below is the link to the electronic supplementary material.

Supplementary file1 (DOCX 175 KB)

## Data Availability

The data that support the findings of this study are available on request from the corresponding author, [CRR]. The data are not publicly available due to their containing information that could compromise the privacy of research participants.

## Abbreviations

AE: Adverse event
BPIC-SS: Baseline bladder pain/interstitial cystitis symptom score
EoT: End of treatment
ESSIC: European Society for the Study of Interstitial Cystitis
FAS: Full analysis set
GAG: Glycosaminoglycan
HMW: High molecular weight
IC/BPS: Interstitial cystitis/bladder pain syndrome
PGA: Patient global assessment
QoL: Quality of life
SAF: Safety analysis set
UTI: Urinary tract infection
VAS: Visual analog scale
