# Supplementary material for: A prospective, multicenter, real-world effectiveness and safety study of high molecular weight sodium hyaluronate for interstitial cystitis/bladder pain syndrome
Source: Int Urol Nephrol. 2026 Feb 5;58(8):2963–72. doi: 10.1007/s11255-026-05035-1 (PMC13375820; doi:10.1007/s11255-026-05035-1)
Supplement: Supplementary file 1 — Supplementary file1 (DOCX 175 KB) [file 11255_2026_5035_MOESM1_ESM.docx]

**Supplementary Data**

**Table 1. Summary of Pre-treatment Medical History in the SAF (N = 73)**

| **Condition/Disease** | **n (%)** | **Events** |
| --- | --- | --- |
| Medical/surgical/allergy history | 38 (52.1) | 176 |
| Congenital, familial and genetic disorders | 1 (1.4) | 2 |
| Congenital hypercoagulation | 1 (1.4) | 1 |
| Factor XIII mutation | 1 (1.4) | 1 |
| Ear and labyrinth disorders | 1 (1.4) | 1 |
| Hypoacusis | 1 (1.4) | 1 |
| Endocrine disorders | 9 (12.3) | 9 |
| Goiter | 2 (2.7) | 2 |
| Graves’ disease | 1 (1.4) | 1 |
| Hyperthyroidism | 1 (1.4) | 1 |
| Hypothyroidism | 5 (6.8) | 5 |
| Eye disorders | 3 (4.1) | 3 |
| Cataract | 1 (1.4) | 1 |
| Allergic conjunctivitis | 1 (1.4) | 1 |
| Gastrointestinal disorders | 12 (16.4) | 17 |
| Abdominal pain | 1 (1.4) | 1 |
| Chronic gastritis | 1 (1.4) | 1 |
| Constipation | 1 (1.4) | 1 |
| Diverticulum intestinal | 2 (2.7) | 2 |
| Duodenal ulcer | 1 (1.4) | 1 |
| Dyspepsia | 1 (1.4) | 1 |
| Gastritis | 2 (2.7) | 2 |
| Gastroesophageal reflux disease | 1 (1.4) | 1 |
| Hiatus hernia | 4 (5.5) | 4 |
| Inguinal hernia | 1 (1.4) | 1 |
| Irritable bowel syndrome | 1 (1.4) | 1 |
| Large intestine polyp | 1 (1.4) | 1 |
| General disorder and administration site condition | 1 (1.4) | 1 |
| Pain | 1 (1.4) | 1 |
| Hepatobiliary disorders | 1 (1.4) | 1 |
| Hepatic steatosis | 1 (1.4) | 1 |
| Immune system disorders | 1 (1.4) | 1 |
| Hypersensitivity | 1 (1.4) | 1 |
| Infections and infestations | 7 (9.6) | 8 |
| Injury, poisoning, and procedural complications | 2 (2.7) | 2 |
| Investigations | 5 (6.8) | 7 |
| Metabolism and nutrition disorders | 15 (2.0.5) | 16 |
| Musculoskeletal and connective tissue disorders | 9 (12.3) | 12 |
| Neoplasms benign, malignant, and unspecified (incl. cysts and polyps) | 5 (6.8) | 6 |
| Nervous system disorders | 3 (4.1) | 3 |
| Pregnancy, puerperium, and perinatal conditions | 1 (1.4) | 1 |
| Psychiatric disorders | 12 (16.4) | 13 |
| Renal and urinary disorders | 4 (5.5) | 5 |
| Lower urinary tract symptoms | 1 (1.4) | 1 |
| Micturition urgency | 2 (2.7) | 2 |
| Renal cyst | 1 (1.4) | 1 |
| Urethral caruncle | 1 (1.4) | 1 |
| Reproductive system and breast disorders | 9 (12.3) | 10 |
| Respiratory, thoracic, and mediastinal disorders | 11 (15.1) | 12 |
| Skin and subcutaneous disorders | 6 (8.2) | 7 |
| Social circumstances | 2 (2.7) | 2 |
| Surgical and medical procedures | 12 (16.4) | 28 |
| Vascular disorders | 9 (12.3) | 9 |

**Abbreviations:** n, number of patients in group; N, total number of patients in the population; SAF, safety set.

**Table 2. Summary of Patients with IC/BPS with Concomitant Pain Medications**

| **Medications by therapeutic class or generic name** | **SAF (N = 73)** |
| --- | --- |
| ≥1 Concomitant medications±, n (%) | 13 (17.8) |
| Analgesics | 5 (6.8) |
| Metamizole | 1 (1.4) |
| Paracetamol | 2 (2.7) |
| Pregabalin | 1 (1.4) |
| Tramadol | 1 (1.4) |
| Anti-inflammatory and anti-rheumatics | 5 (6.8) |
| Dexketoprofen Trometamol | 2 (2.7) |
| Ibuprofen | 2 (2.7) |
| Naproxen | 1 (1.4) |
| Neurologicals‡ | 6 (8.2) |
| Amitriptyline | 4 (5.5) |
| Amitriptyline Hydrochloride | 1 (1.4) |
| Botulinum Toxin Type A | 2 (2.7) |

**Abbreviations:** IC/BPS, interstitial cystitis/bladder pain syndrome; n, number of patients in group; N, total number of patients in the population; SAF, safety set; VAS, visual analog scale

±Concomitant medications were medications that were started after enrollment in the study. ‡Patients may have taken more than one medication within a therapeutic class or generic name. Patients were only counted once for each drug class or generic name summary.

**Table 3. Summary of Treatment Response Results from Prior Studies**

| **Author, Year** | **Study Design** | **# of patients** | **Instillation Schedule** | **Response Rates** |
| --- | --- | --- | --- | --- |
| Morales et al., 1996 | Uncontrolled | 25 | Weekly for 4 weeks followed by monthly for one year | 71% |
| Porru et al., 1997 | Uncontrolled | 10 | Weekly for 6 weeks | 30% |
| Leppilahti et al., 2002 | Prospective cohort | 11 | Weekly for 4 weeks | 64% |
| Kallestrup et al., 2005 | Prospective cohort | 20 | Weekly for one month, followed by monthly for two months | 65% |
| Daha et al., 2005 | Prospective cohort | 48 | Weekly; 40 mg of hyaluronic acid for 10 consecutive weeks | 89% |
| Gupta et al., 2005 | Prospective cohort | 36 | Weekly; for 6 weeks | 55% |
| Riedl et al., 2008 | Prospective cohort | 121 | Weekly | 85% |
| Lai et al., 2012 | Prospective, randomized | 30 | Weekly for 4 weeks followed by five monthly, versus biweekly for 12 weeks | 69%-73% |
| Reidl & Morales, 2018 | Prospective randomized placebo-controlled trial | 137 | Weekly for 8 weeks | 61% |

**Figure 1. Patient Global Assessment Scale for Assessing Treatment Response**


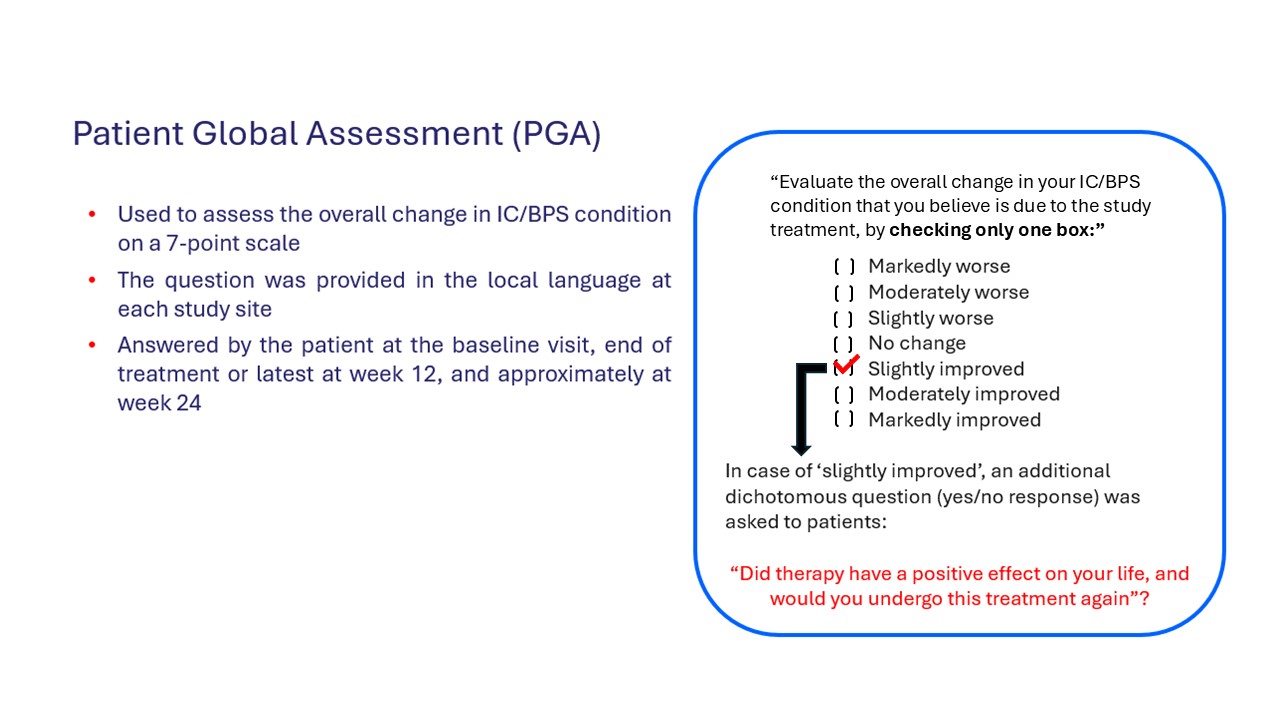
**Abbreviations:** IC/BPS, interstitial cystitis/bladder pain syndrome


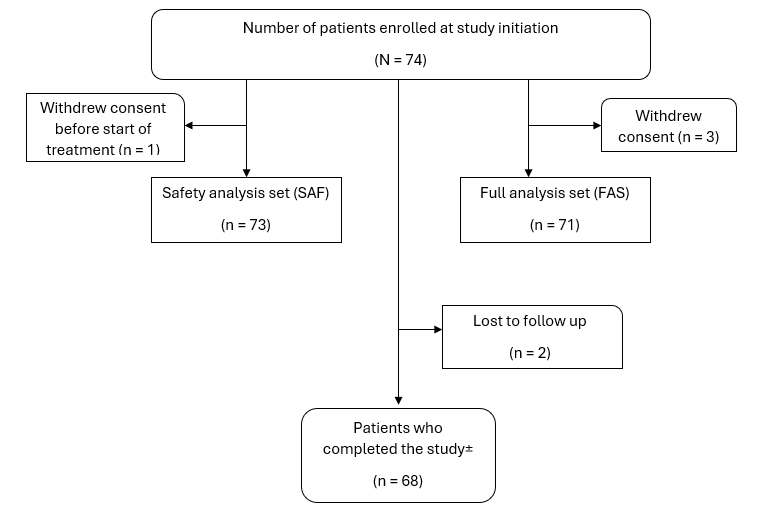
**Figure 2. Patient Disposition**

**Abbreviations:**  FAS, full analysis set; n, number of patients in group; N, total number of patients in the population; SAF, safety set.

±Possible reasons for not completing the study could be withdrawal of consent by patients, diagnosis of pregnancy or becoming pregnant during the study, loss to follow up, and discretion of the investigator if it was deemed to be in the patient’s best interests to terminate the study treatment.
